# Beyond the status-quo: research on Bayesian reasoning must develop in both theory and method

**DOI:** 10.3389/fpsyg.2015.00097

**Published:** 2015-02-06

**Authors:** Simon J. McNair

**Affiliations:** Centre for Decision Research, Leeds University Business School, University of LeedsLeeds, UK

**Keywords:** Bayesian reasoning, individual differences, cognition, psychological methods, subjective probability

Judgements in the real-world often inherently involve uncertainty, from the mundane: “do those clouds signal rain?” to the potentially life-changing: “Does this person have cancer?” Normatively estimating the likelihood of outcomes in such situations involves considering how competing sources of probabilistic evidence (“how likely are clouds with/without rain?”) should be weighed against prior probabilities (“how likely is it to rain/not rain?”), known as *Bayesian reasoning*. This complex form of reasoning, however, typically eludes many people, and can have dramatic implications including overdiagnosis (e.g., Casscells et al., 1978), and wrongful conviction (e.g., the famous Sally Clark case in the UK. See Nobles and Schiff, 2007). Whilst the question of how best to assist people to make such judgments remains in critical need of research (e.g., Navarrete et al., 2014), this paper considers how extant research on Bayesian facilitation has been somewhat constrained by both theoretical, and methodological status-quos. As Mandel (2014) notes, in more general terms we still know relatively little about “what it is to ‘be Bayesian’,” which has clear implications for our understanding of “what works and why” in Bayesian intervention. This paper contemplates several suggestions as to how research may improve its pursuit of this goal, including the deconstructing of Bayesian reasoning into component tasks, and the leveraging of more process-oriented measures to further integrate burgeoning findings concerning individual cognitive differences.

Although research has discovered several interventions that can facilitate more accurate Bayesian judgments, discussion has centered on a distinct division as to the psychological basis of these facilitation effects. Facilitation is often explained as being due to either (a) humans having evolved a cognitive primacy specifically for naturally sampled data (e.g., Gigerenzer and Hoffrage, 1995; Brase, 2009), or alternatively (b) an activation of more general analytical cognitive processes through explicating nested subset relations (e.g., Sloman et al., 2003; Yamagishi, 2003). Whilst the former, evolutionary hypothesis advocates facilitation through expressing data as natural frequencies, the latter, nested-sets hypothesis argues that reasoning can be improved irrespective of numerical format by generally clarifying set relations in the structure of the available evidence, such as through the use of visual diagrams. The debate between both positions, to a large extent, continues to define the literature on Bayesian reasoning (more recently Brase, 2008; Hill and Brase, 2012; Lesage et al., 2013; Sirota et al., 2014). But, whilst there continues to be disagreement on how best to facilitate Bayesian reasoning, one might look to the research and note the distinct variability in reported improvements produced by both frequency- and set-based interventions.

To illustrate, uncertain data expressed as naturally-sampled frequencies can increase Bayesian accuracy as high as either 76% (Cosmides and Tooby, 1996), 54% (Evans et al., 2000), or 31% (Sloman et al., 2003) where equivalent measures have been used. Similarly, equivalent visual diagrams that elucidate nested set relations, irrespective of numerical format, can improve accuracy rates as high as 80% (Yamagishi, 2003), 48% (Sloman et al., 2003), or 35% (Brase, 2009). Such variability exposes a particular limitation common to both perspectives in that neither theory offers satisfactory explanations as to why many people are seemingly *not* facilitated by their respective interventions. This perhaps stems more generally from the fact that both perspectives provide little specification of the actual mental journey people undergo when attempting to reason in Bayesian terms. By more clearly characterizing what distinguishes those who are and those who are not facilitated we might overcome some of these theoretical limitations and, ultimately, further extend our understanding of how best to improve Bayesian reasoning beyond the theoretical divide that currently exists.

Approaching this issue involves a slight shift in perspective from “what works and why?” in Bayesian facilitation to “what works for whom, and why?” (see Hill and Brase, 2012; McNair and Feeney, in press, for examples), and more recent research has begun to illuminate a diverse range of psychological capacities associated with Bayesian facilitation. Abilities such as numeracy (e.g., Johnson and Tubau, 2013; McNair and Feeney, in press; though see also Hill and Brase, 2012); cognitive reflection (Lesage et al., 2013); and fluid intelligence (e.g., Sirota et al., 2014) have variously being associated with good Bayesian reasoning, which may go some way in explaining why previous research has noted such variability in facilitation findings (see Brase et al., 2006, for related concerns). Yet, identifying that component abilities and traits are associated with facilitation effects answers only part of the above question. Moreover, recent discussion of individual differences in Bayesian facilitation has remained grounded in the evolutionary and nested-sets debate as it stands, and as such there exists limited extrapolation of these findings beyond the abstract activation of either a frequency-processing engine in the brain, or set-based analytical processing [though see discussions of Sirota et al. (2014) and Johnson and Tubau (2013) for some further speculation]. Of further interest is exactly how these individual differences in facilitation are manifest in terms of differential thought processes that separate good Bayesian reasoning from bad.

Other recent research, for instance, is beginning to unearth exactly how different cognitive abilities inform different forms of reasoning (e.g., Del Missier et al., 2013). Elsewhere, De Neys and Bonnefon (2013) consider that cognitive individual differences may occur either early or late in the reasoning process. Their contention is that early individual divergences in the reasoning process may represent a more fundamental lack of formal knowledge, whilst later divergences may represent failures in appropriately applying knowledge. Given this hypothesis, individual differences in facilitation effects could be leveraged to signal the particular step in the Bayesian process on which a particular intervention exerts most benefit. For this type of approach to yield maximum insight, however, requires more than a slight shift in theoretical perspective; it will also require a reappraisal of some typical methodological practices used in the study of Bayesian reasoning.

Mandel (2014) succinctly notes several issues that have typified the archetypal methods used to study Bayesian reasoning, notably that of using word problems such as Eddy's (1982) mammography problem. Whilst the use of word problems can provide a convenient litmus test of one's capacity for Bayesian thought, they are often studied in ways that afford limited insight into reasoners' thinking. Two longstanding issues in particular can be identified that, if addressed, would complement attempts to understand how reasoners conduct the process of Bayesian reasoning, and how component abilities map onto this process.

Firstly, word problems predominantly focus on the endpoint of the judgment process, that is: whether someone produces the correct numerical estimate or not. We might conceive of the process of Bayesian judgment as akin to navigating a maze: there is usually one correct path to the exit, but several dead ends that one may arrive at before identifying the correct path. The process of Bayesian reasoning, for most people, may involve a similar process of cognitive tribulation before one reaches the point of arithmetic computation. Yet, by focusing on the endpoint we learn little about the journey. In doing so, research eschews potential opportunities to gain richer awareness into how interventions may change peoples' mental journey through the Bayesian maze, awareness that would further clarify the manner in which these interventions are effective. Future research, then, should look to study *how* reasoners reach their final Bayesian judgments, rather than simply what that final judgment is. One suggestion would be to make greater use of think-aloud protocols to identify the steps at which non-Bayesian deviations occur, and what such deviations entail. Whilst think-aloud paradigms are not without issue—verbalizing thoughts when reasoning can be cognitively challenging (Wilson, 1994); and the mere act of thinking aloud can reactively alter the reasoning process (e.g., Ericsson and Simon, 1998)—the process has previously yielded useful inferences into the types of thoughts underlying errors in Bayesian reasoning (De Neys and Glumicic, 2008). Potential procedural issues are also not without remedy. Although asking reasoners to think-aloud whilst solving more complex Bayesian word problems may prove overly-taxing for the average person, an alternative approach might see the Bayesian task broken down into component steps such as, for instance, information selection; information integration; and finally calculation (see Krynski and Tenenbaum, 2007, for a similar conceptualization). Reducing the overall task into component subtasks presented sequentially may reduce the overall burden of a think-aloud paradigm in this context, and more importantly maximize insight into the exact points in the Bayesian maze at which people deviate from the normative path, permitting more fine-grained interpretations. Varying the think-aloud procedure between subjects should also control for any concern regarding whether a think-aloud approach might actually alter how people would otherwise think about and reason through the task.

A second longstanding issue concerns how research often denotes participant estimates as “correct” (i.e., Bayesian) or “incorrect” (i.e., all other responses). Focusing on the accuracy of judgments alone may conceivably mean an indeterminate number of respondents are perhaps harshly categorized as poor Bayesian reasoners on account of failing to compute a strictly normatively accurate estimate. McNair and Feeney (2013), for instance, observed negligible levels of Bayesian responding on a mammography problem when only exactly arithmetically correct responses were accepted, yet consistently observed that a quarter of all responses fell within 5% of the correct estimate. Furthermore, the specific errors people produce offer potentially rich insights as to how the final judgment was conceived (e.g., Gigerenzer and Hoffrage, 1995); an overly conservative judgment connotes a very different thought process to a wildly inflated estimate. Future research may look to leverage Zhu and Gigerenzer's (2006) “write-aloud” procedure, as an example, which not only identifies a range of discrete errors—each characterized by different reasoning—but also precludes those who produce marginally incorrect estimates as being classified as de facto poor reasoners. Furthermore, rather than dichotomizing responses—which may give a diminished sense of an intervention's effectiveness—reporting *graded improvements* in accuracy (e.g., number of judgments within 5, 10, or 15% of the arithmetic estimate etc.) may also provide an altogether more rigorous evaluation of an intervention's capacity for facilitation.

Research on Bayesian facilitation continues to be productive, as evidenced by the recent upturn in research on individual differences in facilitation effects. Facilitating Bayesian reasoning, ultimately, requires an understanding of the “cognitive tools” people need in order to make such judgments (Ayal and Beyth-Marom, 2014), and how these are applied when engaging in the mental process of Bayesian reasoning. What do people do when navigating the Bayesian maze? At what “step” in the process do deviations from the normative path occur, and are such errors predicted by particular cognitive limitations? The developing picture regarding cognitive capacities and Bayesian reasoning represents an ideal opportunity to more-closely address such questions, but in doing so research must do more to resist certain tendencies that have become somewhat ingrained into the study of Bayesian reasoning. Overcoming these status-quos stands to further elevate our understanding of “what works and why” in Bayesian facilitation through providing greater specifications of the cognitive minutiae involved in producing Bayesian judgments than is currently provided by existing theoretical accounts.

Future research should perhaps look to investigate how specific cognitive capacities relate to each component “step” in the Bayesian reasoning process, taking care to also specify the types of errors produced at each stage, and doing more to distinguish good reasoning and bad arithmetic. The use of more process-oriented methods, such as those considered earlier, can afford a much greater level of fidelity in achieving these goals, and will offer greater insight into what it means to “be Bayesian”—how reasoning progresses; and how, when, and why it sometimes falters. It follows that such research will allow for more targeted refinements in our understanding of what types of intervention strategies may apply best in facilitating better judgments in domains such as health, law, policy, and finance.

## Conflict of interest statement

The authors declare that the research was conducted in the absence of any commercial or financial relationships that could be construed as a potential conflict of interest.
